# ggbio: an R package for extending the grammar of graphics for genomic data

**DOI:** 10.1186/gb-2012-13-8-r77

**Published:** 2012-08-31

**Authors:** Tengfei Yin, Dianne Cook, Michael Lawrence

**Affiliations:** 1 Department of Genetics, Development and Cell Biology, Iowa State University, Ames, IA 50011, USA; 2 Department of Statistics, Iowa State University, Ames, IA 50011, USA; 3 Department of Bioinformatics, Genentech, 1 Dna Way South San Francisco, CA 94080, USA

## Abstract

We introduce ggbio, a new methodology to visualize and explore genomics annotations and high-throughput data. The plots provide detailed views of genomic regions, summary views of sequence alignments and splicing patterns, and genome-wide overviews with karyogram, circular and grand linear layouts. The methods leverage the statistical functionality available in R, the grammar of graphics and the data handling capabilities of the Bioconductor project. The plots are specified within a modular framework that enables users to construct plots in a systematic way, and are generated directly from Bioconductor data structures. The ggbio R package is available at http://www.bioconductor.org/packages/2.11/bioc/html/ggbio.html.

## Rationale

Visualization is an important component of genomic analysis, primarily because it facilitates exploration and discovery, by revealing patterns of variation and relationships between experimental data sets and annotations. Data on the genome fall into two classes: annotations, such as gene models, and experimental measurements, such as alignments of high-throughput sequencing data. The unique and unifying trait of all genomic data is that they occupy ranges on the genome. Associated with the ranges is usually multivariate meta-information both at the feature level, such as a score or functional annotation, and at the sample level, such as gender, treatment, cancer or cell type. These data ranges can range in scale from hundreds to billions of data points, and the features are dispersed along genomes that might be many gigabases in length. Visualization tools need to slice and dice and summarize the data in different ways to expose its different aspects and to focus on different resolutions, from a sensible overview of the whole genome, to detailed information on a per base scale. To help focus attention on interesting features, statistical summaries need to be viewed in conjunction with displays of raw data and annotations.

Various visualization tools have been developed, most of which are implemented in the form of a genome browser. Data are typically plotted along with annotations with genomic coordinates on the horizontal axis with other information laid out in different panels called tracks. Examples of genome browsers include the desktop-based browsers Integrated Genome Browser [1,2] and Integrative Genomics Viewer [3,4]. There are also web-based genome browsers, including Ensembl [5], UCSC Genome Browser [6], and GBrowse [7], and several new web-based browsers, like Dalliance, which rely on technologies like HTML5 and Scalable Vector Graphics [8], or Adobe Flash, like DNAnexus [9]. Other software, like Circos [10], provide specialist functionality. R also has some new tools for visualizing genomic data, GenomeGraphs [11] and Gviz [12]. They all have advantages for different purposes: some are fast, while others have easier user interfaces. Some are interactive, offer cross-platform support or support more file formats.

Data graphics benefit from being embedded in a statistical analysis environment, which allows the integration of visualizations with analysis workflows. This integration is made cohesive through the sharing of common data models [13]. In addition, recent work on a grammar of data graphics [14,15] could be extended for biological data. The grammar of graphics is based on modular components that when combined in different ways will produce different graphics. This enables the user to construct a combinatoric number of plots, including those that were not preconceived by the implementation of the grammar. Most existing tools lack these capabilities.

A new R package, ggbio, has been developed and is available on Bioconductor [16]. The package provides the tools to create both typical and non-typical biological plots for genomic data, generated from core Bioconductor data structures by either the high-level autoplot function, or the combination of low-level components of the grammar of graphics. Sharing data structures with the rest of Bioconductor enables direct integration with Bioconductor workflows.

## Basic usage

**Figure 1:**
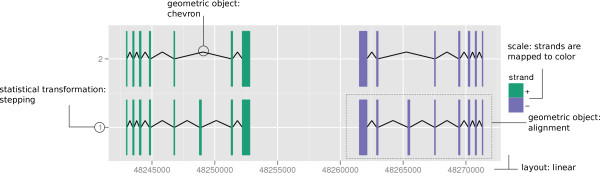
**Gene structure**. Example of exons of gene *SSX4 *and *SSX4B *isoforms, annotated to illustrate the grammar of graphics extensions used. The filled rectangle represents exons and the chevron represents introns. They are grouped by transcript ID and the y axis shows the stepping levels, which stacks transcripts to avoid overplotting. Color is mapped from strand direction.

In ggbio, most of the functionality is available through a single command, autoplot, which recognizes the data structure and makes a best guess of the appropriate plot. Additional file 1 Table S1 lists the type of plot produced for each type of data structure. The plot in Figure 1 was rendered with the autoplot function in ggbio, using the following code:

autoplot(grl,aes(color=strand))

The data, grl, is a GRangesList object, a Bioconductor data structure for representing compound ranges, including a set of transcript structures. The autoplot function recognizes the GRangesList object and draws the intervals in the typical fashion for a gene model or alignment. The y axis is generated by a layout algorithm to ensure that the transcripts are not overplotted. The x axis is automatically set as the genomic coordinates. The call to aes maps the strand variable to the color aesthetic. Users can specify labels, titles, layout, and so on by passing additional arguments to autoplot. Compared to the more general qplot API of ggplot2, autoplot facilitates the creation of specialized biological graphics and reacts to the specific class of object passed to it. Each type of object has a specific set of relevant graphical parameters, and further customization is possible through the low-level API, introduced later.

## Plotting tracks

In many genome visualizations, different datasets are typically plotted separately and then stacked on top of the same x axis, the genomic coordinates. These plots are often called tracks, because they are usually much wider than they are tall and run parallel to each other. Each track might contain a heatmap, or a histogram, or a density plot, for example. The data displayed in each track is related to the data displayed in the other tracks through the shared genomic axis. The goal is to be able to observe different patterns in these snapshots for regions of the genome. When displaying a relatively large region, the tracks might show a summary of the data, whereas more details are depicted for smaller regions.

The ggbio package provides a function called tracks, which stacks plots in a specified order and creates a Tracks object. The object allows users to zoom and shift the plot, as well as modify parameters like the height and theme of individual tracks. In the following example, p1, p2 and p3 are plot objects in the session:

tracks(p1,p2,p3,heights=c(4,1,1))

**Figure 2:**
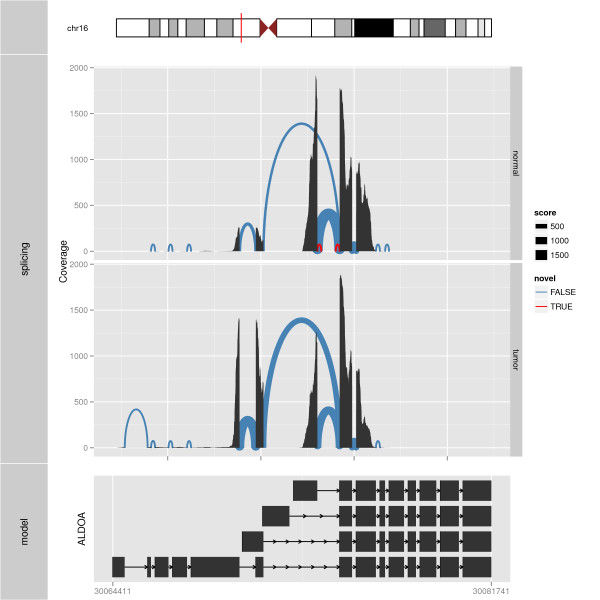
**Splicing summary with coordinate truncate gaps**. An example of a plot made from multiple tracks. At the top, the relevant chromosome is drawn with the subregion of interest marked in red. The middle track shows the slicing summary plots for the gene *ALDOA *for normal and tumor samples. Splicing is shown as arches and size is used to represent junction counts and color represents novelty: blue indicates known splicing events against the model and red indicates novel splicing events. The height of arches is proportional to the distance between the two ends of the arches, or the distance between the junction reads. Coverage is shown by position to address supporting evidence in the raw data. The splicing summary plots are aligned with a view of the gene structure in the bottom track. The thicker rectangle represents the Consensus Coding DNA Sequence (CCDS) transcripts. The plots in both tracks are made with truncate gaps coordinate transformation. The space dedicated to introns has been significantly reduced, and the exonic regions are shown in detail, even though the entire gene region is in view.

Figure 2 illustrates the creation of tracks. One tumor/normal pair of RNA-seq samples is shown with the goal of interpreting splicing changes. A key aspect of the plot is how the read alignment coverage and junction counts are composited and then juxtaposed with the data from the other sample, as well as the annotated transcript models. The viewer is then able to relate the changes in coverage to the corresponding transcript structures, via the common x axis. At the top of the figure is an ideogram overview of the chromosome, using colors corresponding to the Giemsa stain.

## Genomic overviews

The purpose of an overview plot is to give a grand view of the entire genome. By definition, this means that the resolution will be poor and that only large features will be visible. An overview may reveal large features that might be missed if one focused too narrowly. Different methods for mapping the genomic axis to the screen have been applied to address the space issues, and also to ease the drawing of connections between regions.

### Grand linear view

**Figure 3:**
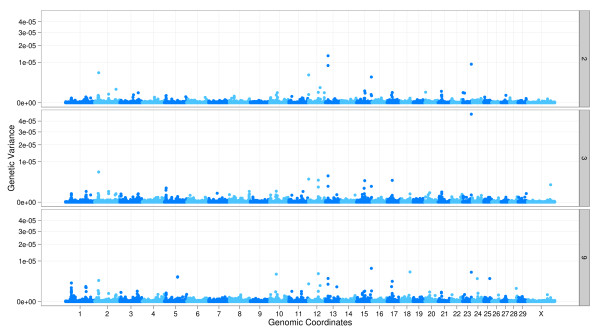
**Manhattan plot**. Grand linear view applied to a Manhattan plot as part of a genome-wide association study in Angus cattle. The y axis shows genetic variance, calculated by sliding windows of five consecutive SNPs for the infectious bovine keratoconjunctivitis (IBK; a type of pinkeye) score. The x axis is the genomic coordinates with all the chromosomes side-by-side. The horizontal striping of color helps to indicate the end of one chromosome and beginning of another. The plot is faceted by three different analysis methods. There is one extreme variance in the middle facet, in the region of chromosome 23. There are also a few large values in other regions. According to the results from the paper, three of these regions, 2, 13, and 23 are found to be potentially indicative of a quantitative trait locus associated with IBK.

Figure 3 shows an example of a grand linear view, which lays out the entire genome along a single linear axis. The plot shown in the figure is a special case of the grand linear plot called a Manhattan plot, due to its resemblance to the Manhattan skyline. It emphasizes extreme events, which show up as high-valued outliers. This view has been used for many genome-wide association study reports [17]. The data here come from a genome-wide association study on Angus cattle, and the data are faceted by three different scoring and classification methods [18]. The horizontal axis shows the global genomic coordinates, and the vertical axis is generally mapped to some quantity of interest, in this case, the genetic variance.

The plot uses linear layout and employs the genome coordinate transformation, which transforms the chromosomal coordinates into global genomic coordinates as if all of the chromosomes were concatenated together. The transformation supports both proportional and uniform scaling of chromosomes, so that the plot area consumed by a chromosome is either proportional to its length or the same as the other chromosomes. It is also possible to add a buffer or break between chromosomes.

### Karyogram overview

**Figure 4:**
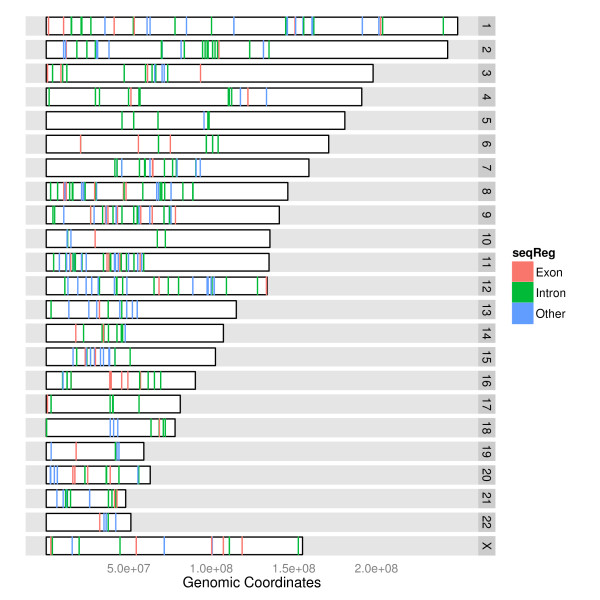
**Stacked karyogram overview**. Karyogram plot shows a subset of human RNA-editing sites, and they are color coded for different regions as follows: red indicates exons, green indicates introns and blue indicates exons/introns status is unknown.

Figure 4 shows a (single copy) karyogram overview plot, with the color indicating RNA-editing locations in human [19]. The karyogram layout represents chromosomes as rectangles and stacks them vertically or in a grid layout. Genomic position is relative to the chromosome, starting from the first position at the left for each rectangle. Associated information is overlaid on or over the box. Applications like Genome Graphs in the UCSC genome browser [6] provide a similar plot. The advantage of this layout over the grand linear view is more efficient use of horizontal space, and hence finer resolution detail on the positions of the features. The trade-off is that the second variable has less space - instead of a full vertical axis the information needs to be fit into each rectangle. Thus, we obtain genomic position resolution at the cost of less data layer resolution. It is common to see this type of plot used for SNP density, varying levels of identity-by-descent (IBD) [20] and length of linkage disequilibrium spans [21].

### Circular overview

**Figure 5:**
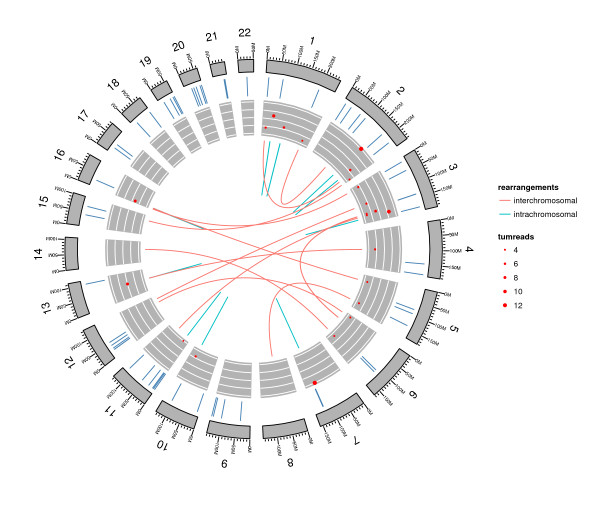
**Single sample circular view**. DNA structural rearrangements and somatic mutation in a single colorectal tumor sample (CRC-1). The outer ring shows the ideogram of the human autosomes, labeled with chromosome numbers and scales. The segments represent the missense somatic mutations. The point tracks show score and support for rearrangement. The size of the points indicates the number of supporting read pairs in the tumor and the y value indicates the score for each rearrangement. The links represent the rearrangements, where intrachromosomal events are colored green and interchromosomal events are colored orange.

The primary purpose of the circular view is to show links between genomic regions. This is generally infeasible with the linear or karyogram layouts. In a circular layout, features are organized into concentric rings. Figure 5 illustrates the circular overview on the data from a gene fusion study conducted by Bass and colleagues [22], who sequenced the genomes of nine individuals with colorectal cancer and identified an average of 75 somatic rearrangements per tumor sample. This circular view shows only a single sample (colorectal tumor sample CRC-1), the structural rearrangements are shown as links with intrachromosomal events in green and interchromosomal translocations in orange. An ideogram of the autosomes is shown in the outer ring, with somatic mutation and score tracks in the plot. There are many software packages that provide a circular overview plot, including Circos [10], CGView [23] and DNAplotter [24].

## Specialized plots

There are some typical types of plots used to examine specific biological questions. This section describes how ggbio builds two of these: a mismatch summary and an edge-linked interval plot.

### Mismatch summary

**Figure 6:**
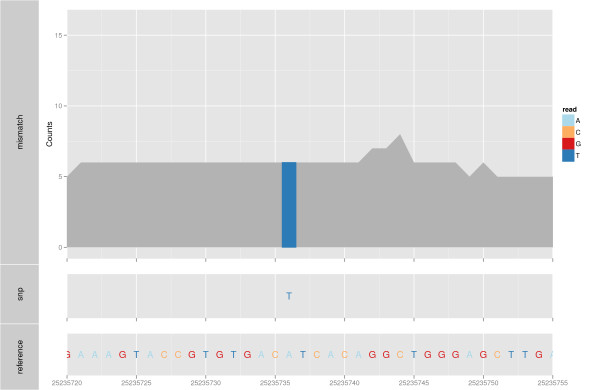
**Mismatch summary**. An example of a mismatch summary plot, with associated variant calls. The top track shows a barchart of reference counts in gray and mismatched counts colored by the nucleotide. The middle track shows SNPs as letters, color coded also by nucleotide. There is one mismatch, 'T', that is different for all of the reads from the 'A' in the reference genome (bottom letter plot).

Mismatch summary is one typical way to visualize alignments from sequencing data, especially in the context of variant calling. Other genome browsers, such as Integrative Genomics Viewer [4], Savant [25] and Artemis [26] render similar plots from BAM and variant call format (VCF) files. Figure 6 shows two different summaries of mismatches from a set of RNA-seq read alignments. The top plot shows one DNA-seq sample from the first phase of the 1000 Genomes Project [27], represented as a stacked barchart. It provides a detailed view of the coverage, where the counts of bases that match the reference are indicated by gray bars, and the counts of non-reference bases are indicated by a different color that is specific to the base (A, C, G or T).

### Edge-linked interval to data views

**Figure 7:**
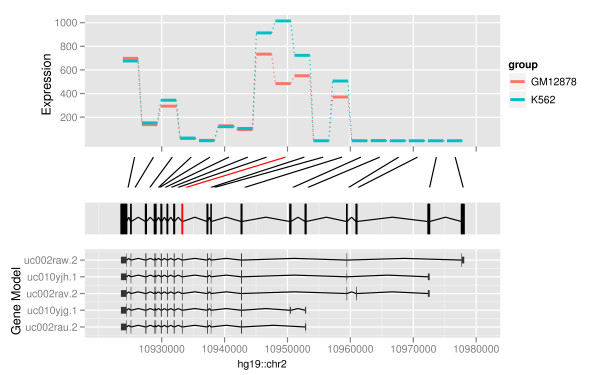
**Edge-linked interval to data view**. Edge-linked interval to data view for the expression of the exons of gene *PDIA6*. The top track shows the expression level for each of the exons, and the color indicates the sample (GM12878 or K562). The second track shows the links between the even-spaced expression track and the exons track, below. The package DEXseq, which produces a similar graphic, computes differential expression and significance, and significance is indicated by coloring the connecting lines red. The track at the bottom shows the annotated transcripts.

Interval data, like genes, regulatory sites, read alignments, and so on, are different lengths. Differences in length can be distracting when looking at associated numerical information. Thus, length is sometimes best ignored, and the interval treated as an id or categorical variable. Figure 7 shows an example. The top plot shows a profile display of expression levels for two samples, GM12878 and K562, where the genomic position of the exons is treated as a categorical variable, forcing equal width in the plot. This allows us to see exons where the expression level is different, without being distracted by the relative interval size of the exons. We could also consider this display to be a parallel coordinate plot [28,29].

## Biological extensions to the grammar of graphics

### The base grammar

**Figure 8:**
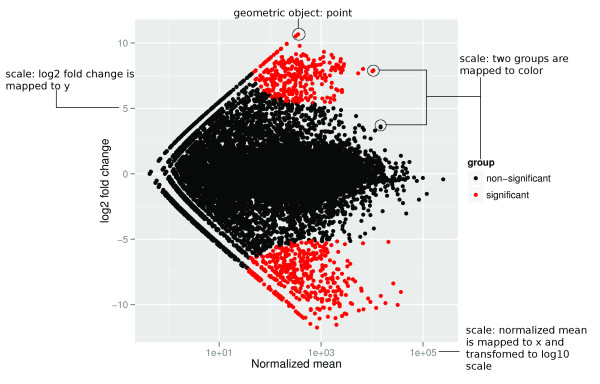
**MA-plot**. MA-plot for differential expression analysis in four RNA-seq samples with two cell lines GM12878 and K562, annotated to illustrate the use of the grammar of graphics. Points is our geometric object, x axis indicates the normalized mean and the y axis indicates the log_2 _fold change. Aesthetics mapping took place between the groups and the color to use red to indicate the most significant differently expressed observation (gene). This plot uses Cartesian coordinates.

The work introduced in this paper builds upon the grammar of graphics conceived by Wilkinson [14] and expanded upon by Wickham [15]. The grammar is composed of interchangeable components that are combined according to a flexible set of rules to produce plots from a wide range of types. Table 1 explains the components of the grammar (data, geom, stat, scales, coord, facet), as utilized in ggplot2, and indicates how these are used to create two graphics: Figure 8 and 1.

**Table 1:** Components of basic grammar of graphics

Comp	Explanation	Figure 8 usage	Figure 1 usage
Data	Data to visualize, containing variables and values	A gene expression table	A GRanges object (core data structure in Bioconductor)
Geom	A geometric object draws the data as a graphical primitive. Types of primitives include points, lines, polygons or text. Some statistical or composite primitives, such as histogram, boxplot and point range, are considered to be geoms	Points with color indicating significance of expression (red = significant, black = not)	Alignments (new), Chevron (new)
Stat	A statistical transformation transforms, filters and/or summarizes a variable prior to plotting. For example, binning and counting is necessary to make a histogram. The default would be an identity transformation, which does not change the data. In ggplot2 an appropriate default transformation is chosen according to the geom, for example, the bin transform for the histogram geom. Thus, the user rarely needs to explicitly specify one	Identity (computation of M value and A values is done outside of the grammar)	Steppings (new)
Scales	A scale maps the variables (for example, expression, treatment, gene id) from data space to aesthetics (for example, position, color, area). Scales also control associated guides like axes and legends. Included in scales are numerical transformations such as log or square root of variables, so that an axis can be drawn on a log scale, for example. The default is a linear scale	A, the log geometric average, the x axis, and M, the log ratio mapped to the y axis	Genomic position mapped to position along x axis, and levels mapped to y axis
Coord	A coordinate system controls how two position scales work together. The default is the Cartesian coordinate system, but others such as a polar coordinate system could be chosen	Cartesian	Cartesian
Facet	A faceting specification is used to produce small multiples [42] for subsets of the data. In other graphical systems it is known as latticing [43], trellising [44] or even conditioning	None	None
Layout (new)	A layout is a new grammatical component for controlling how multiple plots are arranged in a figure. It was motivated by the need to display multiple genomic annotation data sets simultaneously, and also supports genomic overviews	Single	Linear

**Figure 9:**
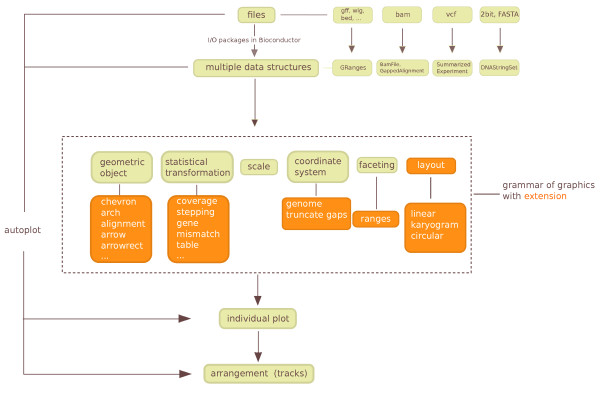
**Diagram of the ggbio framework for processing sequence data**. It starts with a mapping from different file types to different objects or data structure in R, using Bioconductor tools, followed by general and extended grammar of graphics mapping of data elements to graphical components. The final stage arranges the graphics in a designed layout to show annotation tracks or multiple data sets. Orange boxes and dark brown arrows indicate the extensions provided by ggbio.

Components of the basic grammar of graphics, and the extended grammar, and how they are used in Figures 8 and 1. Figure 9 illustrates how the grammar has been extended for biological data. Entries marked with 'new' are those developed as part of this work; the rest are inherited from ggplot2.

### Genomic data and abstractions

Data are the first component of the grammar, and data may be collected in different ways. Wilkinson makes a distinction between empirical data, abstract data and metadata [14]. Empirical data are collected from observations of the real world, while abstract data are defined by a formal mathematical model. Metadata are data about data, which might be empirical, abstract or metadata themselves. We will use the term data source to refer to concrete data in specific databases and file formats. This is roughly analogous to Wilkinson's empirical data.

The ggbio package attempts to automatically load files of specific formats into common Bioconductor data structures, using routines provided by Bioconductor packages, according to Additional file 1 Table S2. The loaded data are then considered by Wilkinson to be abstract, in that they are no longer tied to a specific file format. Analogously, a data structure may be created by any number of algorithms in R; all that matters is that every algorithm returns a result of the same type. The type of data structure loaded from a file or returned by an algorithm depends on the intrinsic structure of the data. For example, BAM files are loaded into a GappedAlignments, while FASTA and 2bit sequences result in a DNAStringSet. The ggbio package handles each type of data structure differently, according to Additional file 1 Table S1. In summary, this abstraction mechanism allows ggbio to handle multiple file formats, without discarding any intrinsic properties that are critical for effective plotting.

### Extension overview

Genomic data have some specific features that are different from those of more conventional data types, and the basic grammar does not conveniently capture such aspects. The grammar of graphics is extended by ggbio in several ways, which are illustrated in Figure 9 and described in Additional file 1 Table S3. These extensions are specific to genomic data, that is, genomic sequences and features, like genes, located on those sequences.

Figure 1 illustrates how the components of the grammar are combined to plot gene structures. A sample of the data is shown in Table 2. The data are passed to ggbio as a GRangesList object. The chevron geom mimics the typical splice junction diagram found in textbooks and it draws the introns in the example. The exons are drawn using the rectangle geom, and the high-level alignment geom ensures that the introns and exons from the same transcript are drawn connected, according to the tx_id column. The position on the chromosome is mapped to the horizontal axis and strand is mapped to color. The vertical axis is mapped to a variable generated by the stepping statistic, which avoids overplotting between the transcripts. We will explain these aspects in the following sections.

**Table 2:** Example of GRanges object

	seqnames	ranges	strand	tx_id	exon_id
1	chrX	[48242968], [48243005]	+	35775	132624
2	chrX	[48243475], [48243563]	+	35775	132625
3	chrX	[48244003], [48244117]	+	35775	132626
4	chrX	[48244794], [48244889]	+	35775	132627
5	chrX	[48246753], [48246802]	+	35775	132628
...	...	...	...	......	...
26	chrX	[48270193], [48270307]	-	35778	132637
27	chrX	[48269421], [48269516]	-	35778	132636
28	chrX	[48267508], [48267557]	-	35778	132635
29	chrX	[48262894], [48262998]	-	35778	132633
30	chrX	[48261524], [48262111]	-	35778	132632

Typical biological data coerced into a data frame: a GRanges table representing genes *SSX4 *and *SSX4B*. One row represents one exon, seqnames indicates the chromosome name, ranges indicates the interval of exons, strand is the direction, tx_id and exon_id are the internal ids used for mapping cross-database.

### Biological geometries

A geom is responsible for translating data to a visual, geometric representation according to mappings between variables and aesthetic properties on the geom. In comparison to regular data elements that might be mapped to the ggplot2 geoms of points, lines and polygons, genomic data has the basic currency of a range. Ranges underlie exons, introns and other features, and the genomic coordinate system forms the reference frame for biological data. We have introduced or extended several geoms for representing ranges and gaps between ranges. They are listed in Additional file 1 Table S3. For example, the alignment geom delegates to two other geoms for drawing the ranges and gaps. These default to rectangles and chevrons, respectively. Having specialized geoms for commonly encountered entities, like genes, relegates the tedious coding of primitives, and makes user code simpler and more maintainable.

### Biological statistical transformations

**Figure 10:**
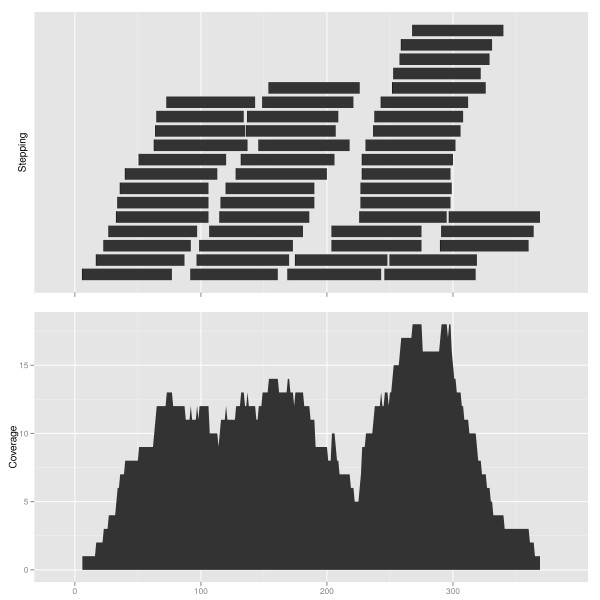
**Coverage transformation**. Statistical transformation, coverage and stepping, are used to summarize short reads data. Top: a set of (simulated) short reads, displayed using the stepping transformation, vertically, and the default geom 'rectangle'. Bottom: coverage is shown on the vertical axis, using the geom 'area'. This example applies the data model GRanges object.

A statistical transformation (stat) transforms or summarizes the data in a particular way. These statistics may be mapped to visual aesthetics in the same manner as the original data. In this work we introduce several statistical transformations specifically for genomic data as shown in Additional file 1 Table S3. For example, given a large number of read alignments, computation of coverage is useful, as shown in Figure 10. These trans-formations were implemented with significant help from Bioconductor tools. New statistical transformations are readily incorporated.

### Biological coordinate transformations

Coordinate systems locate points in space, and we use coordinate transformations to map from data coordinates to plot coordinates. The most common coordinate system in statistical graphics is cartesian. The transformation of data to cartesian coordinates involves mapping points onto a plane specified by two perpendicular axes (x and y). Why would two plots transform the coordinates differently for the same data? The first reason is to simplify, such as changing curvilinear graphics to linear, and the second reason is to reshape a graphic so that the most important information jumps out at the viewer or can be more accurately perceived [14].

Coordinate transformations are also important in genomic data visualization. For instance, features of interest are often small compared to the intervening gaps, especially in gene models. The exons are usually much smaller than the introns. If users are generally interested in viewing exons and associated annotations, we could simply cut or shrink the intervening introns to use the plot space efficiently. For example, Figure 2 is able to show the entire gene region, with virtually no loss in data resolution. In ggbio, we propose three sets of coordinate systems, shown in Additional file 1 Table S3, which are useful for genomic data.

### Biological faceting

Almost all experimental outputs are associated with an experimental design and other meta-data, for example, cancer types, gender and age. Faceting allows users to subset the data by a combination of factors and then lay out multiple plots in a grid, to explore relationships between factors and other variables. The ggplot2 package supports various types of faceting by arbitrary factors. The ggbio package extends this notion to facet by a list of ranges of interest, for example, a list of gene regions. There is always an implicit faceting by sequence (chromosome), because when the x axis is the chromosomal coordinate, it is not sensible to plot data from different chromosomes on the same plot. As an aside, generating a set of tracks might resemble faceting, but it is easier to fit into the grammar framework if we think of it as a post-processing step.

### Biological layout

We have also extended the grammar of graphics with an additional component called layout, upon which the mapping from genomic coordinates to plot coordinates depends. The default layout simply maps the genomic coordinates to the x axis and facets by chromosome. The currently supported layouts are: linear (genomic coordinates mapped to the x axis), karyogram (each chromosome displayed separately, in an array), and circular (like linear, except wrapped around in a circle). The high-level genomic overview plots take advantage of these layout mechanisms.

## Low-level grammar-oriented API

For custom use cases, ggbio provides a low-level API that maps more directly to components of the grammar and thus expresses the plot more explicitly. Generally speaking, we strive to provide sensible, overridable defaults at the high-level entry points, such as autoplot, while still supporting customizability through the low-level API.

All lower level functions have a special prefix to indicate their role in the grammar, like layout, geom, stat, coord, and theme. The objects returned by the low-level API may be added together via the conventional + syntax. This facilitates the creation of new types of plots. A geom in ggplot2 may be extended to work with more biological data model, for example, geom rect will automatically figure out the boundary of rectangles when the data is a GRanges, as do geom bar, geom segment, and so on. As an example, the following code produces the same plot as the code shown above, using the low-level API instead of autoplot:

ggplot()+geom_arrowrect(unlist(grl))+geom_chevron(gaps(unlist(grl)))

The reader will notice how the low-level code is more descriptive about the composition of the plot. In this example, it says we start with an empty plot as created by ggplot. We then use geom arrowrect for exons and add a second layer for the gaps using geom chevron.

The low-level API may be used in conjunction with autoplot, via the + syntax. This makes it possible to save a plot as an object in a session and modify it in different ways while experimenting. For example, the following code applies a new theme to the existing graphic object. The theme null function removes the background labels and legend.

$$\begin{array}{c} \text{p}<-\text{autoplot}\left(\text{grl},\text{aes}\left(\text{color}=\text{strand}\right)\right)\\ \text{p}+\text{theme}\text{\_}\text{null}\left(\right) \end{array}$$

## Materials and methods

The ggbio package is an extension for R, a free cross-platform programming environment for statistical analysis and graphics with more than 3, 000 contributed packages. The package depends upon Bioconductor libraries for handling and processing data, including the implementation of the statistics in our extension of the grammar. The Bioconductor project is a collaborative effort to develop software for computational biology and bioinformatics with high-quality packages and documentation [30]. The visualization methods in ggbio depend heavily on the package ggplot2 [15], which implements the grammar of graphics. The new geoms in ggbio are constructed from primitives defined in ggplot2. We use ggplot2 as the foundation for ggbio, due to its principled style, intelligent defaults and explicit orientation towards the grammar of graphics model. The color schemes in ggbio were derived from standard palettes available in R [31-33].

The RNA-seq data used in this paper are from ENCODE [34]. Two cell lines, GM12878 (blood, normal, female) and K562 (blood, cancer, female), are selected, and there are two replicates for each sample. The data were mapped against hg19 using Spliced Transcript Alignment and Reconstruction (STAR) [35]. The Bioconductor packages Rsamtools [36] and GenomicRanges [37] were used to import the BAM files and count reads overlapping exons. The package DEXSeq [38] was used to conduct the expression analysis and find the most differently expressed exons. We used the rtracklayer package [39] to import BED format files and cast them into GRanges objects for ggbio. The DNA-seq BAM files and VCF files used in Figure 6 were downloaded from the 1000 Genomes Project [27].

All figures, code and data links are available from the documentation section of the ggbio website [40].

## Discussion

We have demonstrated how ggbio supports both the convenient construction of typical genomic plots, while simultaneously supporting the invention of new types of plots from low-level building blocks. Use cases of ggbio range from generating reproducible, exploratory plots in the course of an analysis to the prototyping of new ways of looking at these complex data. Lessons learned might be applied to the design of more complex, interactive systems. A new package, visnab, is being developed to make interactive graphics for genomic data [41].

One such lesson learned is the importance of color choices, which are inconsistent in many existing tools. Color is one of the primary visual clues in a data graphic and needs to be handled with some intelligence. For example, the ggbio package builds on well-specified color palettes used in ggplot2 and biovizBase, including one that is based on the biologically inspired Giemsa stain colors, as shown at the top of Figure 2.

## Abbreviations

API: application programming interface; ENCODE: The Encyclopedia of DNA Elements; SNP: single nucleotide polymorphism; UCSC: University of California Santa Cruz; VCF: variant call format.

## Competing interests

The authors declare that they have no competing interests.

## Authors' contributions

Tengfei Yin and Michael Lawrence designed and wrote the package. Michael Lawrence provided the initial ideas on extending the grammar for biological data. Dianne Cook and Michael Lawrence provided advice on plot design, and suggestions for the design and development of the package. All three authors substantially contributed to the manuscript writing, and have approved the final version.

## Supplementary Material

Additional file 1 **Supplementary Tables**. Table S1: Data model. Table S2: Supported data formats. Table S3: Extension of grammar of graphics. Click here for file
