# Supplementary material for: ggbio: an R package for extending the grammar of graphics for genomic data
Source: Genome Biol. 2012 Aug 31;13(8):R77. doi: 10.1186/gb-2012-13-8-r77 (PMC4053745; doi:10.1186/gb-2012-13-8-r77)
Supplement: Additional file 1 — Supplementary Tables. Table S1: Data model. Table S2: Supported data formats. Table S3: Extension of grammar of graphics. [file gb-2012-13-8-r77-S1.PDF]

Tables

Table S1 - Data model

Object	Usage	Icon
IRanges	basic intervals	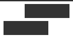
GRanges	genomic intervals	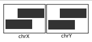
GRangesList	nested intervals	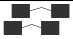
GappedAlignments	gapped alignments	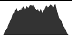
BamFile	read alignments	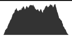
character	external files	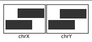
Rle/RleList	genome-length vectors	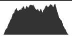
TranscriptDb	gene structure	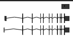
BSgenome	genomic sequence	C T G
VCF	variants	A<br>C T G
ExpressionSet	summarized expression data	
GenomicRangesList	multiple datasets	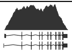

Formal data model and the corresponding default graphics. These are also the supported objects for the `autoplot` function in *ggbio*, which produces the corresponding graphic automatically.

Table S2 - Supported data formats

Data source	Abstract data	Usage
gff, bed, wig, BigWig...	GRanges	genomic regions with meta-data
bam	BamFile<br>GappedAlignments	reference to bam files<br>container for store alignments
vcf	SummarizedExperiments	container for ranges of interests and multiple assay data
2bit, FASTA	DNAStringSet	container for storing a set of nucleotides or amino acids.
sqlite	TranscriptDb	genomic features

Supported abstract data structures (class names) and related data sources.

Table S3 - Extension of grammar of graphics

Component	Name	Usage	Icon
**geom**	geom_rect	rectangle	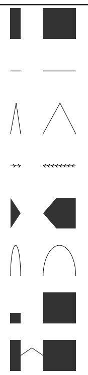
	geom_segment	segment	
	geom_chevron	chevron	
	geom_arrow	arrow	
	geom_arrowrect	arrow	
	geom_arch	arches	
	geom_bar	bar	
	geom_alignment	alignment (gene)	
**stat**	stat_coverage	annotation/read depth	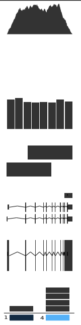
	stat_mismatch	mismatch pileup for alignments	
	stat_aggregate	aggregate in sliding window	
	stat_stepping	avoid overplotting	
	stat_gene	consider full gene structure	
	stat_reduce	consider reduced gene structure	
	stat_table	tabulate ranges	
**coord**	linear	ggplot2 linear but facet by chromosome	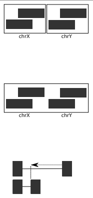
	genome	put everything on genome coordinates	
	truncate gaps	compact view by shrinking gaps	
**layout**	linear	genomic coordinates on x axis	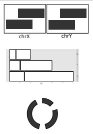
	karyogram	karyogram display	
	circle	circular	
**faceting**	formula	facet by formula	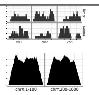
	ranges	facet by ranges	
**scale**	not extended	*ggplot2* default	

Components of the basic grammar of graphics available in *ggplot2*, with the extensions made by *ggbio*. Some of the basic functions from *ggplot2*, recognized because the name is the same, have been extended to work with the biological data models.
